# Prevalence and Related Factors of Low Back Pain in the General Elderly Population: A Japanese Cross-Sectional Study Randomly Sampled from a Basic Resident Registry

**DOI:** 10.3390/jcm10184213

**Published:** 2021-09-17

**Authors:** Masashi Uehara, Shota Ikegami, Hiroshi Horiuchi, Jun Takahashi, Hiroyuki Kato

**Affiliations:** 1 Department of Orthopaedic Surgery, Shinshu University School of Medicine, Nagano 390-8621, Japan; sh.ikegami@gmail.com (S.I.); jtaka@shinshu-u.ac.jp (J.T.); hirokato@shinshu-u.ac.jp (H.K.); 2 Rehabilitation Center, Shinshu University Hospital, Nagano 390-8621, Japan; horiuchih@aol.com

**Keywords:** low back pain, prevalence, influence factor, spinal alignment, aging

## Abstract

Low back pain (LBP) is one of the main etiologies of disability in daily life. In the face of LBP increases in super-aged societies, there are serious concerns of escalating medical costs and deteriorations in the social economy. It is therefore important to identify the factors associated with LBP for prompt preventative and therapeutic measures. This study investigated the prevalence of LBP and the impact of subject-specific factors on LBP development in Japanese community-dwelling older adults. We established eight groups based on age (50’s, 60’s, 70’s, and 80’s) and gender after random sampling from a resident registry. A total of 411 participants (201 male and 210 female) were enrolled for a whole-spine lateral radiographic examination and dual-energy X-ray absorptiometry. All subjects were evaluated for the presence and degree of LBP. We analyzed the impact of clinical factors on LBP using multivariate analysis. Fifty-three (12.9%) participants (23 (11.4%) male and 30 (14.3%) female) were found to have LBP. The prevalence of LBP tended to increase with age, and similar results were found between genders. In univariate analysis, the subject-related factors of the sagittal vertebral axis, pelvic incidence minus lumbar lordosis (PI-LL) mismatch, and aging had significant associations with LBP. PI-LL mismatch was a significant independent factor in multivariate analysis. In conclusion, this study identified LBP prevalence and subject-specific factors on a general population basis. Multivariate analysis revealed PI-LL mismatch as an independent factor associated with LBP in the healthy community-dwelling elderly.

## 1. Introduction

As the elderly rate reached 28% of the Japanese population in 2019 (Ministry of Internal Affairs and Communications, Statistics Bureau, Population Census), it has become of social importance to clarify the impact of aging in order to extend healthy life expectancy. Low back pain (LBP) is one of the main etiologies of disability in daily life [1]. The lifetime prevalence of LBP is reportedly 80% [2] and has been found to increase with age [3]. Furthermore, LBP may cause depression in the elderly, which has a significant impact on quality of life [4]. LBP was shown to be associated with depression both in the elderly and in middle-aged individuals in the prime of their working life [5,6]. Not only does back pain lead to high medical costs, but the economic and social losses from LBP are considered enormous [7]; in the U.S., the financial loss to LBP has been calculated as up to 120 billion dollars yearly [7]. Several risk factors for LBP have been suggested, including old age, occupation, a sedentary lifestyle, obesity, spinal malalignment, pregnancy, and smoking [8,9]. However, those with the strongest influence on LBP onset remain unknown.

In the present population-based study of the elderly in Japan, we adopted random sampling from the basic resident registry of a suburban town to minimize selection bias and obtain cohort data that more closely resembled the general Japanese population. This epidemiological study was coined “the Obuse study”, bearing the name of the cooperating local government. We have employed the Obuse study cohort for research on various musculoskeletal disorders [10,11,12,13,14,15].

Japan is currently facing a super-aged society unparalleled in the world, with serious concerns of escalating medical costs and significant losses in the social economy [16]. Therefore, it has become paramount to identify the factors associated with LBP development for appropriate early action. This investigation aimed to determine the prevalence of LBP in older Japanese adults using the Obuse study cohort and identify the impact of subject-specific factors, including age, sex, body mass index (BMI), lifestyle habits, comorbidities, and spinal alignment.

## 2. Methods

### 2.1. Study Design

Japanese resident cross-sectional study based on a municipal registry.

### 2.2. Settings

This study was conducted at a hospital in the town of Obuse from October 2014 to June 2017.

### 2.3. Bias

In order to minimize selection bias, we randomly selected candidates from the basic town resident registry.

### 2.4. Study Size

Assuming that the frequency of back pain in the comparison group was between 5% and 20%, sample size calculation estimated that 89 subjects per group would provide 80% statistical power (1 minus beta) with an alpha equal to 0.05. After estimating the possible cohort size in consideration of budget, time, and burden on subjects and research staff, we planned to establish eight groups by age (50’s, 60’s, 70’s, and 80’s) and gender (male and female) containing approximately 50 subjects each for a total of at least 400 subjects. 

### 2.5. Data Source

**Figure 1:**
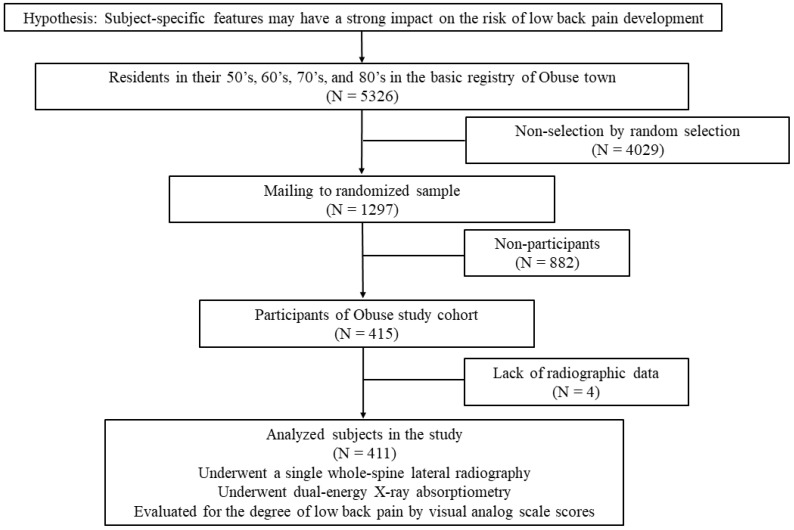
Obuse town resident participant flowchart.

The subject selection process in this study has been previously reported [10]. Briefly, we randomly sampled for candidates from the basic resident registry of the Obuse town (population: 11,326 in 2014). Sampling was conducted until the number of individuals providing consent for study participation reached the target number. A total of 1297 individuals were randomly selected from 5352 people aged between 50 and 89 years in the basic resident registry of the Obuse town in 2014 (Figure 1) [10]. Of them, 882 people were unwilling to participate for undisclosed reasons and excluded from this study. 

### 2.6. Participation

After providing written consent, 415 subjects were enrolled in the Obuse study. The inclusion criteria were residents aged 50–90 years who were randomly selected by town administrative staff from the Obuse resident registry and who consented to participate in the study. The exclusion criteria were subjects with acute LBP, vertebral fracture, spinal infection, or spinal tumor within 3 months prior to the study, as well as those unable to undergo whole-spine radiographs in a standing position. Four people with missing radiographic data were excluded, leaving a total of 411 (201 male and 210 female) Japanese participants. All subjects were measured for physical characteristics and lifestyle habits. The baseline characteristics of the cohort are summarized in Table 1. The protocol of the investigation was approved by our Institutional Review Board (no. 2792). This study was reported in accordance with the STROBE guidelines.

### 2.7. Variables

Analyzed variables included age, gender, height, weight, BMI, smoking, visual analog scale (VAS) score for low back pain, spinal alignment parameters, bone mineral density (BMD), and skeletal muscle mass index (SMI).

### 2.8. Measurement

#### 2.8.1. Measurements of Spinal Alignment

All subjects underwent a whole-spine lateral radiographic examination in a standing position with the hands on the clavicles [17] for the measurement of the sagittal vertical axis (SVA) as an indicator of total spinal alignment as well as pelvic incidence (PI) and lumbar lordosis (LL). A PI minus LL (PI-LL) mismatch was defined as PI-LL >10° [18]. The spinal alignment measurements were performed by 2 board-certified spine surgeons and a trained staff member. The calculated inter-rater reliability scores for each parameter were 0.95 for SVA, 0.80 for PI, and 0.65 for LL [10]. The calculated intra-rater reliability scores for each parameter were 0.91 for SVA, 0.97 for PI, and 0.96 for LL. For validity, our previous study demonstrated that our measurements were comparable to those of previous reports [10]. 

#### 2.8.2. Evaluation of BMD and SMI

All subjects underwent dual-energy X-ray absorptiometry (GE Prodigy, GE healthcare, Chicago, IL, USA) of the lumbar spine. Osteoporosis was defined as a T-score ≤ −2.5 [19]. Skeletal muscle mass was calculated as the sum of the skeletal muscle mass of the arms and legs, assuming that the mass of lean soft tissue was representative of skeletal muscle mass. SMI was calculated as four-limb lean soft tissue mass in kilograms divided by height in meters squared.

#### 2.8.3. Clinical Evaluation of Subjects

All subjects were evaluated for the degree of LBP by VAS scores (0–100 mm). In this study, subjects with moderate to severe LBP, defined as VAS > 50 mm, were considered as having LBP [20]. 

### 2.9. Statistical Methods

Welch’s *t*-test was used to compare the mean values of continuous variables. Fisher’s exact test was adopted to evaluate the differences between categorical variables. We employed a logistic regression model with the existence of moderate or severe LBP (i.e., VAS > 50 mm) as a response variable and subject-specific factor candidates as explanatory variables. Univariate and multivariate analyses using the forced entry method included the factors of sex, BMI, SMI, smoking, BMD, osteoporosis (i.e., T-score ≤ −2.5), SVA >50 mm, PI-LL mismatch, and aging as potential confounding factors of LBP according to previous reports [8,9]. Factors with *p* < 0.2 in the univariate analysis were included in the subsequent multivariate analysis with a stepwise algorithm. All statistical analyses were performed using EZR software (Saitama Medical Center, Jichi Medical University, Saitama, Japan), a modified graphical user interface of R commander (The Foundation for Statistical Computing, Vienna, Austria) designed to add statistical functions frequently used in biostatistics. The level of significance was set at *p* < 0.05.

## 3. Results

### 3.1. Descriptive Data

The prevalence of LBP in the cohort is summarized in Table 2. 

### 3.2. Outcome Data

A total of 53 (12.9%) participants (23 (11.4%) male and 30 (14.3%) female) were found to have LBP among subjects randomly selected from the basic resident registry of a suburb town. There were no cases of acute LBP at the time of data acquisition. The prevalence of LBP for the 50’s, 60’s, 70’s, and 80’s age groups was 6.1%, 5.7%, 14.5%, and 20.0% in men and 17.0%, 6.6%, 14.8%, and 20.8% in women, respectively. The prevalence of LBP tended to increase with age, with the exception of 50’s women. Similar results were observed between genders.

### 3.3. Main Result

In univariate analysis, the subject-specific factors of SVA, PI-LL mismatch, and aging had significant associations with LBP, while those of sex, BMI, BMD, SMI, and smoking did not. PI-LL mismatch was the only significant independent factor according to multivariate analysis, with an odds ratio of 1.91 (Table 3). 

## 4. Discussion

### 4.1. Key Result

This study evaluated the prevalence and related factors of LBP by random sampling from the basic resident registry of a suburb town for subject selection with age and gender clustering on a general population basis. LBP prevalence tended to increase comparably with age for both genders apart from 50’s women, for which social activities and stress were possible reasons for the higher incidence. Multivariate analysis considering various confounders, such as age, gender, and BMI, revealed PI-LL mismatch as an independent factor associated with LBP. These findings may help in the early detection and treatment of LBP in subclinical or asymptomatic community-dwelling members.

Although numerous factors have been linked to LBP, their authenticity remains under debate [8,9]. Several reports have described an association between obesity and LBP [21,22]. In one population-based study, BMI was significantly associated with higher chronic LBP prevalence in women [22]. Hirano et al. also showed BMI to be strongly associated with lumbar spinal canal stenosis in community-living people [23]. On the other hand, Dario et al. witnessed that BMI did not increase the risk of chronic LBP in a population of Spanish adult twin [24]. In another study, although obesity was not associated with overall chronic LBP, its impact was more pronounced for severe chronic LBP [25]. In the present investigation, BMI did not significantly associate with LBP. Social factors and lifestyle have also been cited in relation to LBP, with reports implicating smoking with LBP [26,27]. In contrast, smoking and alcohol were not significantly linked to LBP in a cross-sectional prospective study of young twins [28], with conflicting associations for smoking [29,30]. We observed no remarkable associations for smoking with LBP.

Several reports have described a relationship between BMD and LBP [31,32,33]. A small-sample study argued that lower BMD of the lumbar spine was more frequent among LBP patients and that LBP could increase the risk of osteopenia [31]. On the other hand, another report found that participants with LBP had significantly higher lumbar BMD than did those without LBP, concluding that the presence of rotational asymmetry and associated motion restriction increased BMD in affected vertebrae [32]. A population-based cross-sectional study also showed an association for lumbar BMD with LBP [33]. In this investigation, however, BMD was not significantly related to LBP.

Regarding the influence of muscle mass, paraspinal muscle volume has been linked to sagittal spinal alignment [34,35,36]. Hori et al. described that trunk muscle mass was significantly associated with VAS scores in LBP patients visiting spinal outpatient clinics [37]. A systematic review showed that the cross-sectional area of the multifidus muscle was negatively related to LBP, with conflicting evidence for associations between the erector spinae, psoas, and quadratus lumborum cross-sectional area and LBP [38]. Our study found no significant impact for SMI on LBP.

In recent years, corrective surgeries for sagittal spinal deformity have been widely performed in older adults since such disorders were associated with impaired walking and mobility, respiratory and digestive symptoms, and LBP [39,40]. Multiple studies have stated that reduced lumbar lordosis is closely related to chronic LBP in adulthood [39,41]. Kitagawa et al. found that subjects with LBP showed significantly larger SVA and smaller LL as compared with the values of subjects without LBP in a study of total knee arthroplasty patients [42]. In our cohort, SVA > 50 mm, PI-LL mismatch, and aging were significantly associated with LBP in univariate analysis, although PI-LL mismatch alone remained associated with LBP in multivariate analysis (odds ratio: 1.91). A PI-LL mismatch is caused by a compensatory failure of the pelvis in spinal sagittal alignment. In a multicenter study of adult spinal deformity patients, a linear regression model demonstrated the threshold radiographical parameter for the Oswestry Disability Index of >40 to be PI-LL of 11° or more [43]. The results of our study suggest that individuals with LBP may more frequently suffer from pelvic compensatory insufficiency in postural abnormalities.

### 4.2. Limitation

The major limitation of this study was the small study group size due to the method of random sampling from a town population, with resource restrictions to 400 patients due to the inclusion of radiographical examination. Other limitations of the current investigation include a possibility of inter-observer bias and cross-sectional design; we are currently planning longitudinal studies to investigate the prevalence changes of LBP over time. Regional characteristics were also a shortcoming of this study as our subjects were sampled from a suburb area. Indeed, although epidemiological surveys are relatively easy in such regions due to less population displacement, there exists the possibility of differences with urban residents. Lastly, as this was a non-compulsory survey, the proportion of people randomly sampled who ultimately participated was less than one third. Since two thirds of candidates declined to enroll, incomplete selection bias could not be completely removed. 

### 4.3. Generalizability

Nevertheless, the Obuse study cohort is presumed to resemble the average Japanese suburb population very closely due to its survey design. 

### 4.4. Interpretation

Our findings showed spinal alignment to be significantly related to LBP onset and suggested that the early detection of lumbopelvic parameter mismatch by whole-spine radiographs might help prevent LBP occurrence, although further studies are warranted.

## 5. Conclusions

Based on data close to that of the general population, this cross-sectional study confirmed that LBP tended to increase with age in both men and women. Moreover, a high PI-LL mismatch was significantly associated with LBP development in the healthy community-dwelling elderly, which might serve as a simple indicator of health risk and aid in the prevention of back problems in this age group.

## Figures and Tables

**Table 1:** Baseline characteristics.

Gender	Age Group	N	Height, cm Mean (SD)	*p*-Value (vs. 50’s)	Weight, kg Mean (SD)	*p*-Value (vs. 50’s)	BMI Mean (SD)	*p*-Value (vs. 50’s)
Male	50’s	49	171.8 (6.0)		67.1 (9.1)		22.7 (2.9)	
	60’s	53	166.7 (4.7)	<0.01	66.9 (7.7)	0.94	24.1 (2.7)	0.01
	70’s	54	163.1 (5.0)	<0.01	59.9 (10.3)	<0.01	22.4 (3.5)	0.68
	80’s	45	160.1 (5.7)	<0.01	57.5 (8.5)	<0.01	22.4 (2.8)	0.54
	Total	201	165.5 (6.8)		63 (9.8)		22.9 (3.1)	
Female	50’s	47	158.1 (4.9)		55.4 (9.0)		22.2 (3.8)	
	60’s	61	152.8 (5.4)	<0.01	52.2 (7.6)	0.06	22.3 (2.8)	0.86
	70’s	54	149.7 (5.3)	<0.01	50.5 (7.9)	<0.01	22.5 (3.2)	0.68
	80’s	48	144.6 (5.9)	<0.01	48.3 (7.9)	<0.01	23.1 (3.3)	0.21
	Total	210	151.3 (7.1)		51.6 (8.4)		22.5 (3.2)	

BMI: body mass index, SD: standard deviation.

**Table 2:** Prevalence of low back pain.

Age Group	Male	*p*-Value (vs. 50’s)	Female	*p*-Value (vs. 50’s)
50’s	6.1% (3/49)		17.0% (8/47)	
60’s	5.7% (3/53)	1	6.6% (4/61)	0.14
70’s	14.5% (8/55)	0.34	14.8% (8/54)	1
80’s	20.0% (9/45)	0.12	20.8% (10/48)	0.79
Total	11.4% (23/201)		14.3% (30/210)	

**Table 3:** Effects of subject-specific factors on low back pain.

Factor	Univariate Analysis		Multivariate Analysis	
	Odds Ratio (95% CI)	*p*-Value	Odds Ratio (95% CI)	*p*-Value
Sex (male)	1.29 (0.72–2.31)	0.39		
BMI	1.05 (0.96–1.15)	0.26		
SMI	1.04 (0.77–1.40)	0.81		
Smoking	0.77 (0.42–1.41)	0.40		
BMD	1.04 (0.33–3.26)	0.95		
Osteoporosis	0.61 (0.08–4.79)	0.64		
SVA >50 mm	2.3 (1.2–4.4)	0.011		
PI-LL mismatch	2.18 (1.22–3.91)	<0.01	1.91 (1.03–3.55)	0.041
Age (vs. 50’s)				
60’s	0.51 (0.19–1.36)	0.18	0.46 (0.17–1.25)	0.13
70’s	1.33 (0.58–3.02)	0.50	1.24 (0.54–2.83)	0.62
80’s	2.01 (0.90–4.50)	0.089	1.49 (0.64–3.48)	0.36

BMI: body mass index, SMI: skeletal muscle mass index, BMD: bone mineral density, SVA: sagittal vertical axis, PI: pelvic incidence, LL: lumbar lordosis.

## Data Availability

The complete database of the cohort can be accessed at the Zenodo repository (doi.org/10.5281/zenodo.5512411).

## References

[1] GBD (2016). 2015 Disease and Injury Incidence and Prevalence Collaborators. Global, regional, and national incidence, prevalence, and years lived with disability for 310 diseases and injuries, 1990–2015: A systematic analysis for the Global Burden of Disease Study 2015. Lancet.

[2] Fast A. (1988). Low back disorders: Conservative management. Arch. Phys. Med. Rehabil..

[3] Caron T., Bransford R., Nguyen Q., Agel J., Chapman J., Bellabarba C. (2010). Spine fractures in patients with ankylosing spinal disorders. Spine.

[4] Calvo Lobo C., Vilar-Fernández J.M., Losa-Iglesias M.E., López-López D., Rodríguez-Sanz D., Palomo-López P., Becerro-de Bengoa-Vallejo R. (2019). Depression Symptoms Among Older Adults with and Without Subacute Low Back Pain. Rehabil. Nurs..

[5] Calvo-Lobo C., Vilar Fernández J.M., Becerro-de-Bengoa-Vallejo R., Losa-Iglesias M.E., Rodríguez-Sanz D., Palomo López P., López D. (2017). Relationship of depression in participants with nonspecific acute or subacute low back pain and no-pain by age distribution. J. Pain Res..

[6] Lopez-Lopez D., Vilar-Fernandez J.M., Calvo-Lobo C., Losa-Iglesias M.E., Rodriguez-Sanz D., Becerro-de-Bengoa-Vallejo R. (2017). Evaluation of Depression in Subacute Low Back Pain: A Case Control Study. Pain Physician.

[7] Dagenais S., Caro J., Haldeman S. (2008). A systematic review of low back pain cost of illness studies in the United States and internationally. Spine J..

[8] Hoy D., Bain C., Williams G., March L., Brooks P., Blyth F., Woolf A., Vos T., Buchbinder R. (2012). A systematic review of the global prevalence of low back pain. Arthritis Rheum..

[9] Johansson M.S., Jensen Stochkendahl M., Hartvigsen J., Boyle E., Cassidy J.D. (2017). Incidence and prognosis of mid-back pain in the general population: A systematic review. Eur. J. Pain.

[10] Uehara M., Takahashi J., Ikegami S., Tokida R., Nishimura H., Sakai N., Kato H. (2019). Sagittal spinal alignment deviation in the general elderly population: A Japanese cohort survey randomly sampled from a basic resident registry. Spine J..

[11] Uehara M., Takahashi J., Ikegami S., Tokida R., Nishimura H., Sakai N., Nakamura Y., Kato H. (2020). Differences in bone mineral density and bone turnover markers between subjects with and without diffuse idiopathic skeletal hyperostosis. Spine.

[12] Ikegami S., Takahashi J., Uehara M., Tokida R., Nishimura H., Sakai A., Kato H. (2019). Physical performance reflects cognitive function, fall risk, and quality of life in community-dwelling older people. Sci. Rep..

[13] Uehara M., Takahashi J., Ikegami S., Tokida R., Nishimura H., Sakai N., Kato H. (2020). Prevalence of diffuse idiopathic skeletal hyperostosis in the general elderly population: A Japanese cohort survey randomly sampled from a basic resident registry. Clin. Spine Surg..

[14] Tokida R., Uehara M., Ikegami S., Takahashi J., Nishimura H., Sakai N., Kato H. (2019). Association between sagittal spinal alignment and physical function in the Japanese general elderly population: A Japanese cohort survey randomly sampled from a basic resident registry. J. Bone Joint Surg. Am..

[15] Uehara M., Takahashi J., Ikegami S., Tokida R., Nishimura H., Kuraishi S., Sakai N., Kato H. (2019). Impact of diffuse idiopathic skeletal hyperostosis on sagittal spinal alignment in the general elderly population: A Japanese cohort survey randomly sampled from a basic resident registry. JBJS Open Access.

[16] Shinohara S., Okada M., Keira T., Ohwada M., Niitsuya M., Aizawa Y. (1998). Prognosis of accidental low back pain at work. Tohoku J. Exp. Med..

[17] Liu Y., Liu Z., Zhu F., Qian B.P., Zhu Z., Xu L., Ding Y., Qiu Y. (2013). Validation and reliability analysis of the new SRS-Schwab classification for adult spinal deformity. Spine.

[18] Merrill R.K., Kim J.S., Leven D.M., Kim J.H., Cho S.K. (2017). Beyond pelvic incidence-lumbar lordosis mismatch: The importance of assessing the entire spine to achieve global sagittal alignment. Glob. Spine J..

[19] Kanis J.A. (1994). Assessment of fracture risk and its application to screening for postmenopausal osteoporosis: Synopsis of a WHO report. WHO Study Group. Osteoporos Int..

[20] Yamada K., Suzuki A., Takahashi S., Yasuda H., Koike T., Nakamura H. (2015). Severe low back pain in patients with rheumatoid arthritis is associated with Disease Activity Score but not with radiological findings on plain X-rays. Mod. Rheumatol..

[21] Dario A.B., Ferreira M.L., Refshauge K.M., Lima T.S., Ordoñana J.R., Ferreira P.H. (2015). The relationship between obesity, low back pain, and lumbar disc degeneration when genetics and the environment are considered: A systematic review of twin studies. Spine J..

[22] Dario A.B., Ferreira M.L., Refshauge K., Sánchez-Romera J.F., Luque-Suarez A., Hopper J.L., Ordoñana J.R., Ferreira P.H. (2016). Are obesity and body fat distribution associated with low back pain in women? A population-based study of 1128 Spanish twins. Eur. Spine J..

[23] Hirano K., Imagama S., Hasegawa Y., Muramoto A., Ishiguro N. (2013). Impact of spinal imbalance and BMI on lumbar spinal canal stenosis determined by a diagnostic support tool: Cohort study in community-living people. Arch. Orthop. Trauma Surg..

[24] Kakihana H., Jinnouchi H., Kitamura A., Matsudaira K., Kiyama M., Hayama-Terada M., Muraki I., Kubota Y., Yamagishi K., Okada T. (2020). Overweight and hypertension in relation to chronic musculoskeletal pain among community-dwelling adults: The circulatory risk in communities study (CIRCS). J. Epidemiol..

[25] Dario A.B., Loureiro Ferreira M., Refshauge K., Refshauge K., Luque-Suarez A., Ordoñana J.R., Ferreira P.H. (2017). Obesity does not increase the risk of chronic low back pain when genetics are considered. A prospective study of Spanish adult twins. Spine J..

[26] Schembri E., Massalha V., Spiteri K., Camilleri L., Lungaro-Mifsud S. (2020). Nicotine dependence and the International Association for the Study of Pain neuropathic pain grade in patients with chronic low back pain and radicular pain: Is there an association?. Korean J. Pain.

[27] Shiri R., Karppinen J., Leino-Arjas P., Solovieva S., Viikari-Juntura E. (2010). The association between smoking and low back pain: A meta-analysis. Am. J. Med..

[28] Hestbaek L., Leboeuf-Yde C., Kyvik K.O. (2006). Are lifestyle-factors in adolescence predictors for adult low back pain? A cross-sectional and prospective study of young twins. BMC Musculoskelet. Disord..

[29] Esquirol Y., Niezborala M., Visentin M., Leguevel A., Gonzalez I., Marquié J.C. (2017). Contribution of occupational factors to the incidence and persistence of chronic low back pain among workers: Results from the longitudinal VISAT study. Occup. Environ. Med..

[30] Hashimoto Y., Matsudaira K., Sawada S.S., Gando Y., Kawakami R., Kinugawa C., Okamoto T., Tsukamoto K., Miyachi M., Naito H. (2017). Obesity and low back pain: A retrospective cohort study of Japanese males. J. Phys. Ther. Sci..

[31] Gaber T.A., McGlashan K.A., Love S., Jenner J.R., Crisp A.J. (2002). Bone density in chronic low back pain: A pilot study. Clin. Rehabil..

[32] Snider K.T., Johnson J.C., Degenhardt B.F., Snider E.J. (2011). Low back pain, somatic dysfunction, and segmental bone mineral density T-score variation in the lumbar spine. J. Am. Osteopath. Assoc..

[33] Lee S., Nam C.M., Yoon D.H., Kim K.N., Yi S., Shin D.A., Ha Y. (2013). Association between low-back pain and lumbar spine bone density: A population-based cross-sectional study. J. Neurosurg. Spine.

[34] Hiyama A., Katoh H., Sakai D., Tanaka M., Sato M., Watanabe M. (2019). The correlation analysis between sagittal alignment and cross-sectional area of paraspinal muscle in patients with lumbar spinal stenosis and degenerative spondylolisthesis. BMC Musculoskelet. Disord..

[35] Yagi M., Hosogane N., Watanabe K., Asazuma T., Matsumoto M., Keio Spine Research Group (2016). The paravertebral muscle and psoas for the maintenance of global spinal alignment in patient with degenerative lumbar scoliosis. Spine J..

[36] Ferrero E., Skalli W., Lafage V., Maillot C., Carlier R., Feydy A., Felter A., Khalifé M., Guigui P. (2020). Relationships between radiographic parameters and spinopelvic muscles in adult spinal deformity patients. Eur. Spine J..

[37] Hori Y., Hoshino M., Inage K., Miyagi M., Takahashi S., Ohyama S., Suzuki A., Tsujio T., Terai H., Dohzono S. (2019). ISSLS PRIZE IN CLINICAL SCIENCE 2019: Clinical importance of trunk muscle mass for low back pain, spinal balance, and quality of life—A multicenter cross-sectional study. Eur. Spine J..

[38] Ranger T.A., Cicuttini F.M., Jensen T.S., Peiris W.L., Hussain S.M., Fairley J., Urquhart D.M. (2017). Are the size and composition of the paraspinal muscles associated with low back pain? A systematic review. Spine J..

[39] Glassman S.D., Bridwell K.H., Dimar J.R., Horton W., Berven S., Schwab F. (2005). The impact of positive sagittal balance in adult spinal deformity. Spine.

[40] Pellisé F., Vila-Casademunt A., Ferrer M., Domingo-Sàbat M., Bagó J., Pérez-Grueso F.J., Alanay A., Mannion A.F., Acaroglu E., European Spine Study Group (ESSG) (2015). Impact on health related quality of life of adult spinal deformity (ASD) compared with other chronic conditions. Eur. Spine J..

[41] Djurasovic M., Glassman S.D. (2007). Correlation of radiographic and clinical findings in spinal deformities. Neurosurg. Clin. N. Am..

[42] Kitagawa A., Yamamoto J., Toda M., Hashimoto Y. (2021). Spinopelvic Alignment and low back pain before and after total knee arthroplasty. Asian Spine J..

[43] Schwab F.J., Blondel B., Bess S., Hostin R., Shaffrey C.I., Smith J.S., Boachie-Adjei O., Burton D.C., Akbarnia B.A., Mundis G.M. (2013). International Spine Study Group (ISSG). Radiographical spinopelvic parameters and disability in the setting of adult spinal deformity: A prospective multicenter analysis. Spine.

